# Sociocultural and indigenous practices of rural India in adapting to heat stress: an exploratory descriptive qualitative study

**DOI:** 10.1093/inthealth/ihaf153

**Published:** 2025-12-26

**Authors:** Suseendar Shanmugasundaram, Pritish Baskaran, Rehana Vanaja Radhakrishnan, Pankaja Raghav, Anil Kumar Gupta

**Affiliations:** Indian Council of Medical Research – National Institute of Epidemiology, Chennai, India; Department of Community Medicine, Sri Manakula Vinayagar Medical College and Hospital, Puducherry, India; Department of Community Medicine, Andaman and Nicobar Islands Institute of Medical Sciences, Sri Vijaya Puram, Andaman and Nicobar Islands, India; Department of Community Medicine and Family Medicine, All India Institute of Medical Sciences, Jodhpur, India; Integrated Centre for Adaptation, Resilience & Sustainability (ICARS: GOI-DST CoE), Indian Institute of Technology Roorkee, Gr. Noida Campus (NCR), Noida, India

**Keywords:** coping mechanisms, heat stress, indigenous practices, rural India

## Abstract

**Background:**

Rajasthan, a state in northwest India, experiences extreme heat, causing a plethora of health impacts. Understanding local practices for coping with heat is crucial for integrating with available scientific evidence and thereby in developing effective public health interventions. This study aimed to explore the sociocultural practices for adapting to heat stress and health-seeking behaviour for heat-related illnesses among the general population.

**Methods:**

This was an exploratory descriptive qualitative study. In-depth (n=11) and key informant (n=5) interviews and focus group discussions (n=3) were conducted among various stakeholders, with a pretested interview guide. Typical case and intensity sampling techniques were used to recruit participants. Thematic analysis of the content was done manually by an inductive approach to identify various strategies in the community for adapting to heat stress.

**Results:**

A total of 36 participants were involved in this study. They described a range of signs and symptoms experienced during extreme heat. There were mentions of several home remedies, including the use of tamarind paste, bentonite clay, decoction of spices and screw pine flowers. Sociocultural practices for heat adaptation included housing modifications, such as thatched roofs, use of mud and hay, coating with limestone and alum, installation of solar panels and the use of coolers for temperature control. Vocational adjustments, such as scheduling work during cooler times of the day and using wet clothes for heat relief, were also prevalent, with concerns about sickness absenteeism and loss of wages. People expressed their care for animals during the hot summers and nature conservation. Socio-economic disparities within the village are a major reason for challenges related to affordability. Criticisms were also placed on accessibility to a well-prepared health system.

**Conclusions:**

The findings highlight the diverse range of sociocultural practices and health-seeking behaviours by people in rural areas of Rajasthan to cope with extreme heat. Incorporating indigenous knowledge and traditional practices into public health initiatives, after adequate validation, is essential for enhancing heat resilience and mitigating heat-related health risks in the region. Future research and interventions should consider context-specific approaches to heat adaptation, acknowledging the importance of local traditions and practices in promoting community health and well-being.

## Introduction

Heat is a major issue arising from extreme weather conditions, with significant impacts on both human and animal health. Heatwaves are among the most dangerous of natural hazards, but they rarely receive adequate attention because their death tolls and destruction are not always immediately obvious.^1^ Between 2000 and 2019, there were >489 000 heat-related deaths each year, with >70 000 of those deaths occurring during Europe’s 2003 heatwave.^1^ Because of the swift climate change happening across the globe, more people are being exposed to heat. It has been noted that the frequency, duration and magnitude of extreme temperature events are increasing. The number of people who experienced heatwaves has risen by around 125 million between the years 2000 and 2016.^1^

Countries with poor infrastructures are most affected. India is one of the countries in which heatwaves are an emerging concern, especially during recent times.^2^ There are 23 heat vulnerable states spread across the geographic extent of the country, and Rajasthan, in the northwestern part of India, is the one of the most affected states.^3^ Between 1992 and 2015, heatwaves caused 24 223 deaths across the country.^4^ Between 2015 and 2022, India reported 3812 deaths due to heatwaves.^5^

On 19 May 2016, the temperature soared to 51.0°C at Phalodi, one of the districts in Rajasthan.^6,7^ This set a new record for the highest observed maximum temperature ever recorded in the country (and third highest across the globe), with 16 heat-related deaths and increased hospital admissions in the entire state on that day.^7^ More recently, on 25 May 2024, Phalodi once again experienced a searing 50°C during a widespread heatwave, marking the highest temperature recorded in the country since 1 June 2019, when Churu reached 50.8°C.^5^

Rajasthan is identified as one of the regions with the greatest climate sensitivity, maximum vulnerability and lowest adaptive capacity. It faces high risks from climate change impacts, including droughts, heatwaves and water scarcity.^8^

An increase in the core body temperature above a critical threshold can cause a variety of ailments, from non-life-threatening symptoms ranging from heat rash/miliaria, heat oedema, heat cramps, heat syncope and heat exhaustion to life-threatening heat-stroke. People should prepare for a climate-resilient way of living that, even with a small investment, will prevent morbidity and mortality.

However, the underdiagnosis of heat-related illnesses (HRIs) presents a multifaceted challenge rooted in several factors. One significant contributor is the limited preparedness and capacity to effectively manage these conditions. Many healthcare systems lack adequate resources and training to promptly recognize and address HRIs. Furthermore, traditional practices and interventions utilised in various communities are often overlooked or disregarded in modern medical settings. Exploring these traditional approaches may unveil valuable insights and strategies for preventing and managing HRIs. Moreover, there is a pressing need for the integration of traditional practices with scientific evidence to establish comprehensive and culturally sensitive approaches to diagnose and treat HRIs. Efforts aimed at bridging the gap between traditional knowledge and modern scientific methods hold promise for improving the recognition and management of HRIs, ultimately reducing its underdiagnosis and associated health burdens.

It is crucial for scientists and policymakers to take note of traditional practices by people who have lived in such extreme climatic circumstances for multiple generations. The present study aims to explore the practices of the community in the prevention and management of HRIs, alongside their perceptions and health-seeking behaviours towards increasing heat stress and health system preparedness.

## Methods

### Study design and duration

This was an exploratory descriptive qualitative study that was a part of a larger project designed to empower the community for prevention and management of HRIs. This qualitative phase of the study was conducted between March and June 2023. The study was conducted in two villages of different administrative blocks in Jodhpur district, Rajasthan, India.

### Climate of Rajasthan and its vulnerability

**Figure 1.**
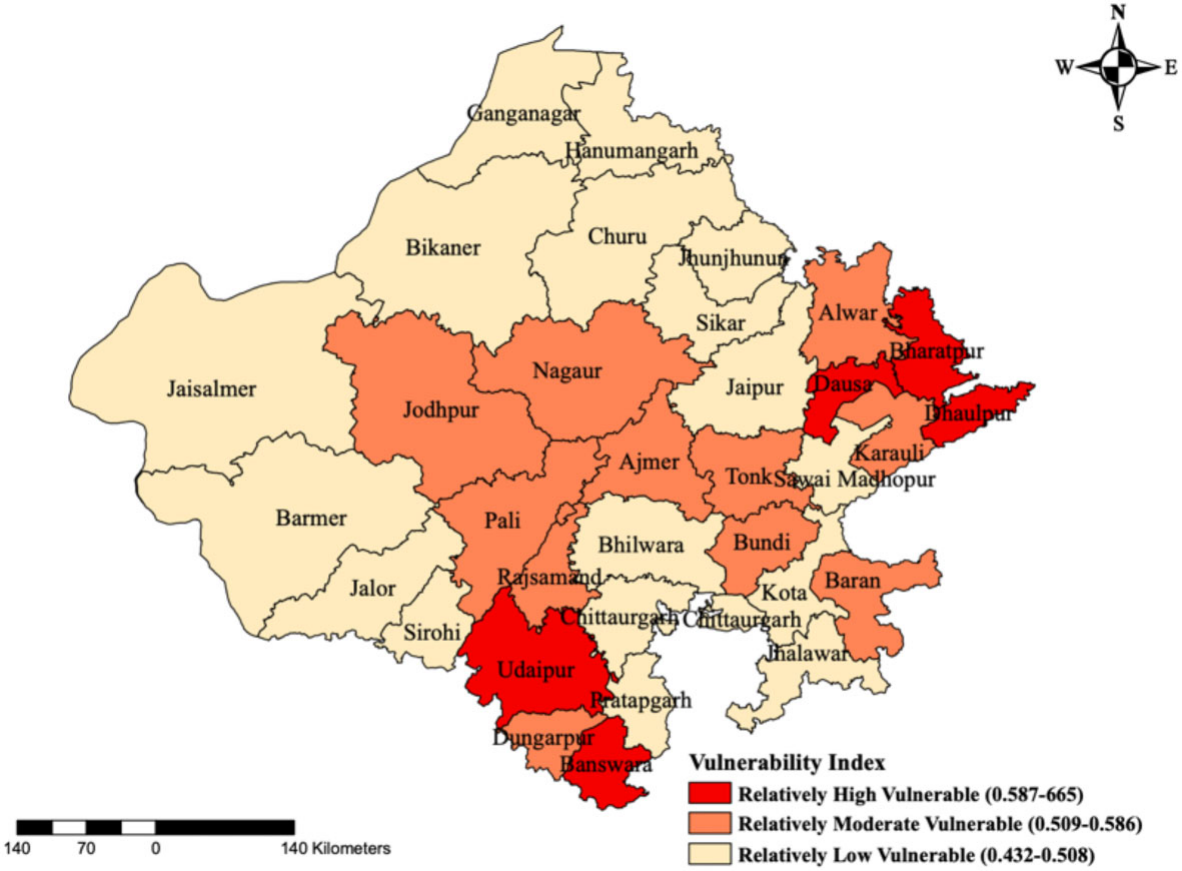
Map showing the climate vulnerability index of districts in Rajasthan. (Reproduced from Climate Vulnerability Assessment for Adaptation Planning in India Using a Common Framework (2020)^10^)

The state of Rajasthan has a climate that varies from extremely arid to being moderately humid. The humid zone spans the southeast and eastern parts of the state. Except in the hilly areas (the Aravalli Range), the heat during summer is intense everywhere, with temperatures in June (the warmest month) typically rising above 40°C every day (Figure 1). The desert regions in the western part of the state experience extreme climatic conditions. The climate fluctuates from freezing winters to scorching summers, where temperatures can reach up to 50°C, with a brief rainy season between July and September bringing 100–500 mm of rainfall.^9^

Jodhpur, a district situated within the Thar Desert, has a moderate climate vulnerability index, with a score of 0.53. Comparatively, Dholpur has the highest vulnerability index, at 0.665, while Barmer records the lowest at 0.432.^10^

### Study participants and tools

The study included the general population ≥18 years of age, working and non-working members of the household and key informants such as village administrative officers, village leaders and medical officers, among others. Participants were purposively selected (intensity and typical case sampling) based on their knowledge and experience with traditional practices related to heat stress adaptation in Rajasthan. An interview guide was developed (both in English and Hindi) based on a thorough review of the literature and by consultation with experts in the field. The interview guide focused on exploring the perception of the participants on increasing heat in recent years, their traditional or customary practices to tackle heat (while at home, going to work, travel), dietary modifications and agrobiodiversity during summers, infrastructural changes at the household level or at the workplace, health system preparedness and history of tragic events in the village due to heat stress. Interviews were conducted in person, according to the availability and preferences of the participants, and were audio recorded after obtaining their informed consent.

### Data collection and thematic analysis

The participants were introduced to the purpose of the study. Informed written consent for the interview and its recording were obtained. Interviews were conducted by investigators and trained research staff in the vernacular language. Three focus group discussions, 11 in-depth interviews and 5 key informant interviews were conducted (the verbatims and codes are attached as a Supplementary file), following which saturation was attained. The interviews lasted for about 40 min on average and the focus group discussions lasted for about 1 h. Audio recordings and notes were taken during the interviews. Audio recordings were subsequently transcribed verbatim on the same day and translated into English. Qualitative thematic content analysis was performed manually through an inductive approach and preliminary coding schemes were developed. Coding was performed independently by the authors (SS, RVR and PB) and then together to maintain reliability. The reliability of the coding was cross‑verified by all the authors. These coding schemes facilitated the identification of patterns. A data-driven thematic analysis was adopted using a semi-grammatical coding approach.

## Results

### Participant characteristics

The various stakeholders included in the study are presented in Table 1. A total of 36 participants were included in the study, of which 15 were male and 21 were female. Medical officers in different primary and secondary health centres were interviewed, mostly in relation to preparedness of health facilities. The village *sarpanch* (the elected head of the village council) helped in understanding the available amenities and infrastructure and also the age-old traditions in the village. The villagers explained their day-to-day routines, scheduling of work, transport challenges and food habits. Frontline workers emphasized their difficulties in mediating between health systems and the community. The findings of the study are presented under various themes and subthemes (Table 2 and Supplementary Figure 1).

**Table 1.** Description of the stakeholders in the study.

Stakeholders	Males, n	Females, n	Total, N
Villagers	10^a^	12^a^	22
Village *sarpanch* (the elected head of the village council)	3		3
Frontline health workers (ASHAs, ANMs, AWWs)		6^a^	6
Medical officers	2	3	5
Total	15	21	36

ASHA: accredited social health activist; ANM: auxiliary nurse midwife; AWW: Anganwadi workers.

a Including the stakeholders in the focus group discussions.

**Table 2.** Themes and subthemes that emerged through content analysis.

Subthemes	Individual level	Family level	Community level
***Perception about heat-related illnesses***			
Physical symptoms and misconceptions	Dizziness, vomiting, headaches were the most frequent complaints		Misconceptions related to symptoms—referring to any episode of diarrhoea as ‘cholera’ disease
Home remedies	Preference for home remedies for milder illnesses	Bentonite clay (*multhani mitti*) Home-made fluids—lemon water, buttermilk, lassi, boiled vegetable water	
Awareness about heatwaves and alerts	Aware of various channels of communication (young adults)		Informal group called ‘Sajag’ to disseminate weather-related information
***Environmental adaptations***			
Housing adaptations	Avoid cooking in warmer hours of the day Use fossil fuel–based stoves outside the house	Thatched roofs, mud and hay over walls Additional windows Coating floor and roof with alum and limestone Wet sacks or cloths hung over doors and windows	Solar panels over public facilities Temporary shelters and huts Water reservoirs or pots at public places
Protection of animals and nature		Sheds made of straw *chappra* (easy to water, maintains cooler temperature)	Concerns of strays and rabidness Tree plantation drives Temporary shelters and water sources for animals
***Lifestyle and daily activities***			
Vocational and transport adjustments	Scheduling of work to avoid hotter times Carrying water bottles (covered by wet jute) during travel		Disseminating information related to heatwaves to enable scheduling of work Making public transport available during early morning and late evening
Dietary practices	Personal preferences for buttermilk, curd, onions and flatbreads Avoiding meals during hotter hours of the day	Use of *sangri* (desert beans) and dessert like *rabdi* Use of *kevala* (screw pine) flowers in drinking water	Promoting locally available fruits and vegetables such as the watermelon, muskmelon, bananas, mangoes
***System-level challenges***			
Issues with water and electricity		Hire water tankers for domestic purposes	Gram Panchayat arranges for water tankers when water supply from lake is cut off during summers
Issues with healthcare services		Prefer to seek remedial measures	Non-availability of staff at healthcare facilities

### Theme 1: Perception about HRIs

#### Subtheme 1: Physical symptoms and misconceptions

Participants described a range of symptoms experienced during episodes of extreme heat in Rajasthan. Dizziness with or without vomiting was the most prevalent complaint, often accompanied by headaches. Participants frequently reported a burning sensation in their eyes and the soles of the feet, indicative of intense heat exposure. Episodes of infrequent diarrhoea or changes in the consistency of stools were also commonly cited as symptoms during periods of extreme heat. Some villagers, especially very young adults, denied or mentioned that they had not experienced or heard any stories related to heat illnesses or heat-stroke.

There were also some misconceptions about the effects of heat and how the villagers commonly referred to diarrheal episodes as ‘cholera disease’, which required further probing to understand.

“*Cholera disease (diarrhoea) is very common among the old persons. If there is no arrangement for cooler or A/C for them, then it becomes difficult for them even for a day, and sometimes it even leads to death if they go out of the house.*” — Anganwadi helper

Although the villagers perceived the health impacts related to heat stress, they were unable to associate it with when the symptoms occurred.

#### Subtheme 2: Home remedies

Participants shared insights into their health-seeking behaviours when struck with heat stress symptoms. A prominent theme was the self-reliance on home remedies, deeply ingrained in local cultural practices. Tamarind water, which was alleged to possess cooling properties, was frequently consumed or its paste was applied over the skin to alleviate symptoms of heat stress. Bentonite clay, referred to as *multhani mitti*, was also suggested to be applied over the body to have a cooling effect.

“*In earlier days, when there was anybody who was affected with heat-related illness, we used to apply ‘multani mitti’ (bentonite clay) over the body, along with a ground bitter-gourd drink, if they are conscious.*” — An aged male villager

Similarly, oral rehydration solution (ORS) and other oral fluids such as lemon water, buttermilk, lassi (curd with sugar or salt) and boiled vegetable water were cited as traditional remedies for replenishing electrolytes and hydration lost due to excessive sweating. Some participants mentioned the use of a decoction of spices (cloves, coriander, black pepper and sugar) to be fed to patients with heat stress.

“*We make decoctions by adding sugar candy, coriander, black pepper and cloves.*” — An aged female villager in a household)

 

“*…we have curd and buttermilk, we have tamarind water, we drink it by mixing ice cubes (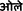)…*”

#### Subtheme 3: Awareness about heatwaves and alerts

The respondents perceived increasing heat over the years, with some pointing to an increasing frequency of sporadic rains in the summer months. While the young adult respondents were aware of most channels (mobile SMS, radio, newspapers, news channels in television) through which heat alerts are provided by the government, the older persons in the village were not aware and also believed that such information could be known only if they have access to smartphones.

The key informant (village *sarpanch*) mentioned about their informal group called *Sajag/*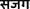 (meaning ‘alert’/‘aware’), which they use to convey and disseminate important information happening in and around the village. These messages are targeted to reach every person in the village, though its members are primarily workers, labourers and shopkeepers.

“*…we get updates from the Rajasthan Meteorological Department and receive official notices predicting increased heat in the next few days. In response, we adjust our MNREGA working hours accordingly. We also inform village residents to stay hydrated during heatwaves. We have a group named ‘Sajag’ to disseminate information to our Gram Panchayat. With social media, everyone is quickly informed. For instance, if a young person sees a weather update at home, they inform everyone. We also rely on TV channels and other media for updates, and people often share this information through statuses and messages…*”

### Theme 2: Environmental adaptations and practices

#### Subtheme 1: Housing adaptations

Participants described housing modifications tailored to withstand the harsh climate, such as the use of thatched roofs to deflect direct sunlight and the incorporation of additional windows to enhance ventilation. The widespread use of air coolers was also highlighted as a popular adaptation strategy, providing respite from the high temperatures.

Older respondents from the village recalled using mud and hay to construct the walls and roofs, which had a cooling effect inside the house. Some also mentioned the use of alum or limestone in coating the floor and roofs to maintain cooler temperatures inside the shelter.

“…when we lived in the kutcha houses*, the roofs were made of mud and hay, which kept the house cooler than the outside environment.” — An old person in a household

 

*Kutcha houses are those that have the roofs made of bamboo, clay, cow dung and/or other naturally available materials.

 

“*…the roofs were separate, and the houses were kutcha, due to which it remained cool in the summer months. Now the roofs are made of tin sheets, which get hot quickly, and are also being made of concrete, making it a closed structure.*”

 

“*If you have a thatched roof, open windows, coat the roof with limestone or alum, it remains cool.*”

The village *sarpanch* (head) stated that installation of solar panels over the roofs of the administrative buildings in the village was planned, and he also said that the people of the village are being encouraged to do the same in their houses. Some respondents stated that they hang wet sacks or cloths over the doors and windows to prevent hot air entering the house.

Setbacks in the household are used to place the open stoves, where fossil fuel is used for cooking. When kitchens are placed inside the house, people prefer to cook their foods early in the morning, store it for the afternoon and cook only late in the evening, avoiding hot air inside the house during the warmer hours.

“*We cook food very early in the morning, and in the evening, to avoid hot air from the kitchen inside the house during hotter hours of the day.*”

At the community level, Mahatma Gandhi National Rural Employment Guarantee Act (MNREGA) workers and community members help in setting up temporary shelters, water reservoirs and huts. This happens as a community-led activity under the supervision of the village head and administrative office.

#### Subtheme 2: Protection of animals and nature

Participants emphasized the importance of protecting cattle from heat stress, employing methods such as constructing *chappra* (animal sheds made of straw) and regularly spraying water to cool the animals. Some respondents brought out concerns regarding strays and the anticipated risks of such animals becoming rabid. In a focus group discussion, the participants recalled and took pride in the famous ‘Bishnoi movement’ in protection of nature and trees. Tree planting drives were mentioned as a beneficial response measure against heat stress, and it was also observed that almost all households had huge trees in their setbacks, offering them shade and cooler temperatures.

“*…the people of the Bishnoi community are deeply devoted to wildlife, as you must have also heard of the movements in the past. Additionally, they take measures such as spraying water on animals and using cooler fans for the cattle sheds.*” — A villager in the administrative office

The role of community participation in preventing the adverse events related to heat stress was high, as people informed us about setting up water sources and sheds for animals all by themselves. Participants perceived the importance of having trees in and around the houses and also expressed concern for cutting them down. They also mentioned the role of village administration in encouraging tree planting drives in the village.

“*…villagers themselves organize and create water sources in public places for animals to drink…*”

 

“*Only a few people are interested to plant trees, while others do not participate in it. Last year, the Gram Panchayat had given medicinal plants to every house. It also feels good that you can breathe and experience cool air by sitting under the plant.*”

### Theme 3: Changes in lifestyle and daily activities

#### Subtheme 1: Vocational and transport adjustments

Adaptations in occupational activities emerged as a key theme in the interviews, reflecting the pragmatic approach of individuals to cope with extreme heat. Many participants described scheduling work during the cooler periods of the day, such as early in the morning and late in the evening, to minimize heat exposure. There were also concerns of sickness absenteeism due to extreme heat and loss of wages because of it, making it difficult for daily wagers, labourers and farmers to make ends meet. The frontline health workers adapted to heat by scheduling their work in the early morning or late in the evening.

“*If people don’t work for 15 days in a month, they can’t sustain their livelihood, so they need to work continuously. Only if they work today, the money they earn will support their household tomorrow, meaning that they must work every day, even in the summers. These individuals’ labourers work in the sun. To cope with this, they take measures to minimise exposure to the sun, such as reducing their time in direct sunlight to 5 to 6 hours.*” — Villager

The MNREGA workers found it difficult to schedule their work hours and consequently suffer more with heat stress. However, the key informants, i.e. the supervisor and the *sarpanch* mentioned that they adjust the work timings if a heatwave alert is received from the Meteorological Department.

“*In the summer season, if the regular work time is 8 hours, it is reduced to 4–6 hours. Similarly, the working hours for MNREGA workers are also nearly halved. For instance, if the normal schedule is from 9 a.m. to 5 p.m., it is adjusted to run from 6 a.m. to 11 a.m. or 12 p.m.*”

**Figure 2.**
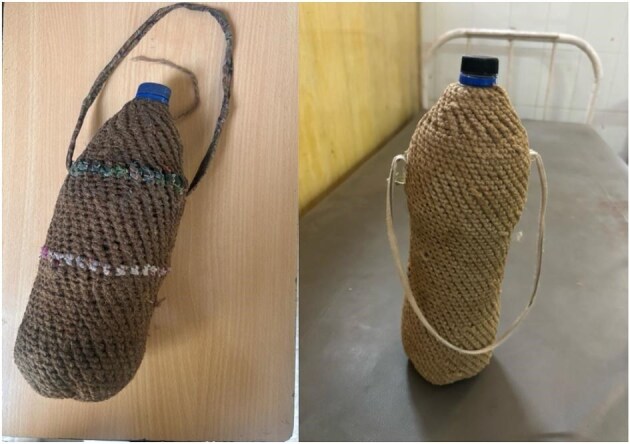
Water bottles wrapped in handmade jute bags, when kept wet, can effectively maintain the cooling of the water inside. This simple yet innovative solution leverages the natural cooling properties of evaporation, providing a sustainable and eco-friendly way to keep water cool in hot climates.

**Figure 3.**
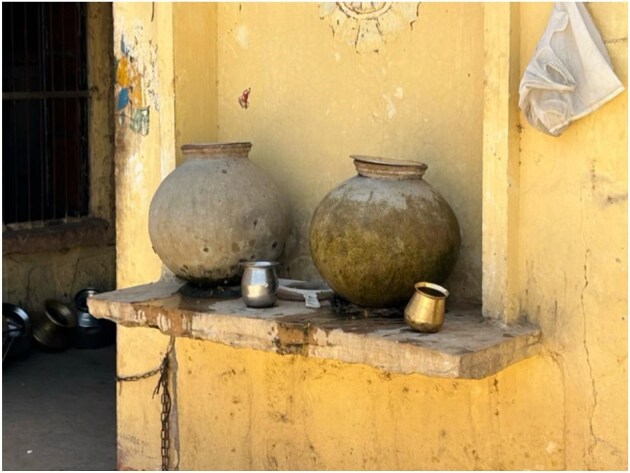
Water pots for drinking in front of most houses, which are accessible to everyone, ensuring hydration for all community members.

Participants elaborated on transportation practices tailored to navigate the challenges posed by extreme heat during travel. Travel arrangements were often strategically planned to coincide with cooler times of the day, such as early morning and evening commutes. Carrying water bottles, sometimes covered by a wet jute sack (Figures 2 and 3), was observed to be a ubiquitous practice to maintain hydration during journeys or even at work.

#### Subtheme 2: Dietary practices

Most of the respondents mentioned the use of *sangri* (desert bean) in their diet during the summer. One of the most common recipes is *Ker sangri*, which is prepared using desert beans and capers.

“*Green vegetables are available here, and dry vegetables are also used like ‘Ker sangri’ (a dish made of desert bean and capers). Apart from this, *rabdi* (a dessert made of thickened milk) made at home is also consumed.*”

People also responded that they preferred more dairy products during the summer, especially curd, condensed milk (*rabri*) and buttermilk. Spicy foods and fried and oily items were reported to be consumed in lesser quantities, as they preferred to consume food prepared with millet, in the form of *rotis* (flat breads) or porridge.

The villagers alleged that the leaves and other parts of the screw pine possessed cooling properties and hence they used it in their water storage pots, which were mostly earthen (Figure 3).

“*Sugar is soaked in water and consumed. ‘Kevala flowers’ (screw pine) are kept soaked in a cold pot, it is considered to cool down the body.*”

 

“*In the summer season, we also use tamarind, soaking it overnight and drinking the water it soaks in, as well as applying it on our feet. Additionally, we consume sugar and use the leaves and flowers of ‘kevala’ (screw pine). We soak ‘kevala’ in water, use its juice, and even bathe with it.*”

People also preferred locally available fruits, such as the watermelon, muskmelon, bananas or mangoes, during the summer, although a considerable number of respondents mentioned they are not affordable.

Throughout the study, it was observed that the socio-economic status of a family and the affordability of commodities played a huge role in their ability to adapt to heat stress. Respondents brought up the issues of affordability while discussing infrastructural changes at the household level or in making dietary changes, and also in adjustments in vocation (sickness absenteeism, loss of wages) or transportation (high dependence on public transport). In contrast, access to an adequate and well-prepared health system in their proximity also posed a great challenge to the villagers, adding to their financial burden in coping with heat illnesses.

### Theme 4: System-level challenges

#### Subtheme 1: Issues with water and electricity

During the summer months, it was reported that there was no regular supply of water, though the demand was higher in the winter months. Participants mentioned that they hired water tankers for their domestic purposes. The problem of water was further accentuated by intermittent power cuts, which lasted for longer hours, and more often occurred during the nighttime. Due to these power cuts, villagers reported that they could not operate motors for agriculture or domestic uses.

“*We hire water tankers, at least two every month. Water is made available once in 10–15 days, sometimes we get it only for one or two hours in a day through the tap. There are also issues with electricity, due to which we are not able to operate the pumping motors and fill waters in the storage tanks. Long hours of electricity cut makes it even harder to store water in adequacy.*”

#### Subtheme 2: Issues with healthcare services

Almost all the respondents mentioned that they seek healthcare at the hospital if the patient does not recover with the initial home remedy given. The study was conducted in a rural setting, near a secondary health facility, and in Anganwadi centres (an initiative of the Ministry of Women and Child Development for providing holistic care to children <5 y of age and pregnant and lactating mothers) near the health facility. Participants expressed grievances against health workers not visiting their homes to ask about their health needs. The frontline workers raised issues about non-availability of staff at health facilities. They also highlighted issues with the availability of healthcare services (drugs, diagnostics) and medical officers and the need to travel to the city for emergencies.

“*There are very limited facilities available here, we usually have to take the patient to the city if there is an emergency.*” — Frontline health worker in the village

**Figure 4.**
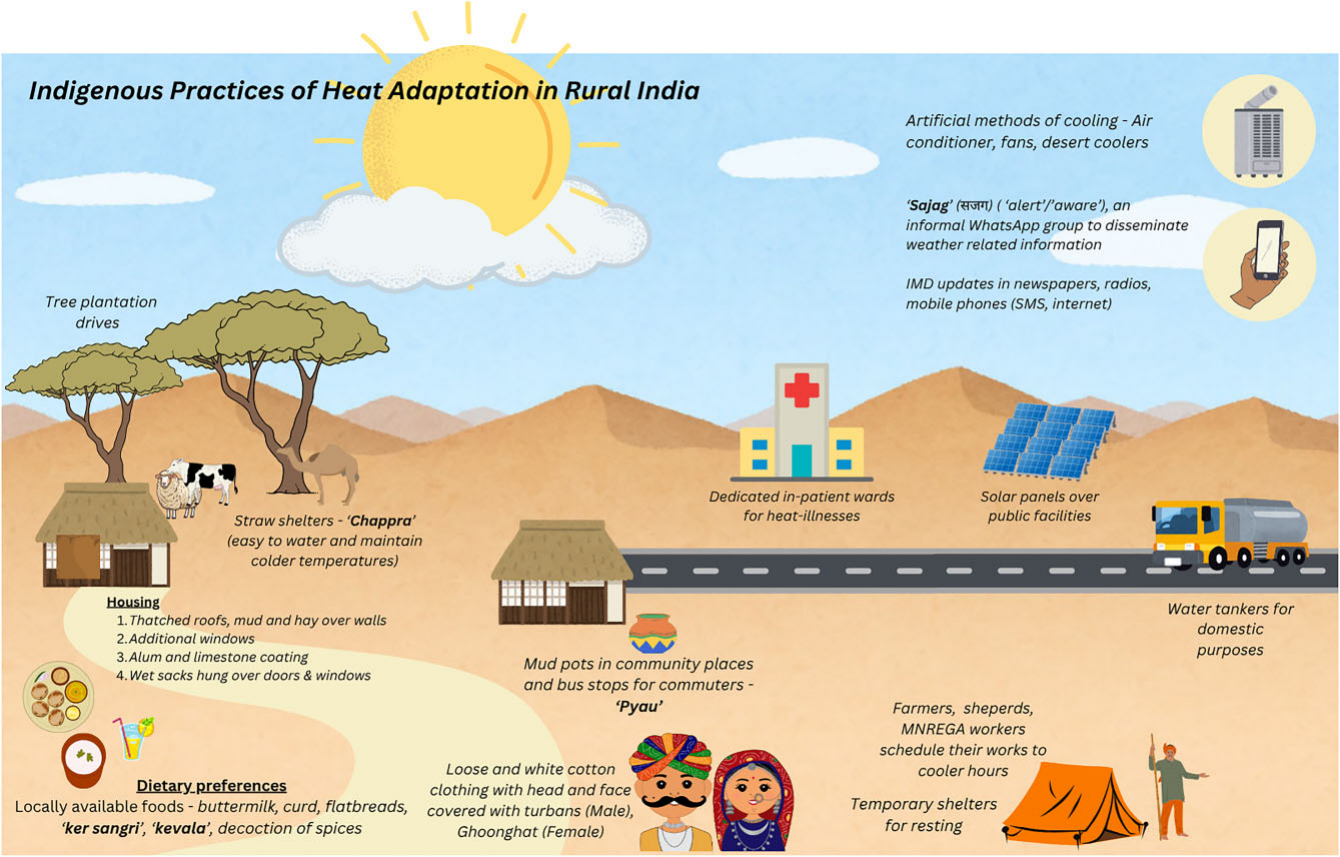
Summary of the important findings from the study.

Figure 4 summarises all the important findings from the study, explaining every category of indigenous practice in mitigating heat stress.

## Discussion

The heat adaptation practices in Rajasthan reveal a robust set of traditional and community-based strategies employed to mitigate the impacts of extreme heat. Previous studies have primarily focused on technological or infrastructural solutions, often overlooking the informal, community-driven adaptations that evolve organically in rural settings. Traditional methods such as consuming tamarind water, using ORS and lemon water and making structural modifications to houses, such as incorporating thatched roofs and additional windows for ventilation were prominently highlighted. Additionally, dietary practices include a preference for local foods and cultivation based on water availability and season, reflecting an adaptation to the harsh climate. These adaptations are being supplemented by community strategies aimed at protecting livestock from heat stress and scheduling work during cooler parts of the day.

*Nautapa* refers to a period of 9 d typically characterized by intense heat (the period when the Earth is closest to sun) in many parts of India, significantly impacting daily life and increasing the risk of heat stress for humans and animals.^11^ Other parts of India also relate to such periods of high temperatures by various names, such as the month of *agni natchathiram* in Tamil Nadu. Hot, dry and sultry months between July and September are referred to as *la canicula* (more commonly known as the ‘dog days’) in the Northern Hemisphere, associated with the constellation of Canis Major (Great Dog) and its star Sirius.^12^ Understanding of these culturally defined days is pertinent, as the people in general are aware of intense heat during this period, for which they are attuned to follow precautions to avoid heat stress.

Participants said that *multani mitti* (bentonite clay) is being traditionally used to alleviate heat stress, due to its alleged natural cooling and soothing properties when applied to the skin. This practice is particularly valuable in regions with extreme temperatures, where it helps reduce discomfort and protect against heat-related illnesses. Evidence suggests that bentonite is being used in sunscreen lotions due to its water resistance and skin adherence properties and a high level of UV absorption compared with sunscreen lotions containing no bentonite.^13^

The decoction made with sugar candy, coriander, black pepper and cloves is a traditional remedy believed to alleviate heat-related symptoms; however, there is no scientific evidence supporting its effectiveness. While experts claim that remedies such as coriander and other herbs can help decrease heat stress due to their cooling properties, the evidence supporting these claims is limited and requires further investigation.

Comparative analysis with other regions of the world underscores the universal relevance and effectiveness of sociocultural adaptation practices. Aboriginal communities in northern Australia, for instance, utilise environmental cues to anticipate and prepare for heatwaves, such as lizards sitting on rocks, ant hills signalling an impending drought, the sky’s appearance early in the morning predicting a very hot day, birds singing in the morning indicating an upcoming hot day and animals behaving differently, like staying hidden, suggesting an approaching drought.^14^ These cues help them prepare for hot weather by moving to areas with reliable water sources and adapting their daily activities to mitigate the effects of the heat. Similarly, in the informal settlements of Dar es Salaam, cost-effective measures such as regular showers, wearing light clothing and opening doors and windows are popular among residents, despite the low use of air conditioners due to issues of affordability.^15^

Eastern India presents a strong preference for nature-based solutions, with trees being a primary coping mechanism for heat stress.^16^ Women, particularly in eastern India, face increased risks due to sociocultural norms that restrict their mobility and expose them to higher indoor heat levels, especially during cooking. This difficulty faced by women when cooking indoors during hot weather is also reflected in this study. One effective intervention involves using well-ventilated kitchens or placing kitchens outdoors to reduce indoor heat exposure. Additionally, shifting from fossil fuels to cleaner energy sources can significantly mitigate indoor heat and improve air quality. The *Ujjwala Yojana* (*Ujjwala* meaning ‘bright’ or ‘radiant’ in Hindi), which provides liquified petroleum gas (LPG) connections to households, is a crucial step in this direction, as it not only reduces indoor air pollution but also helps in lowering heat stress for women, who predominantly handle cooking tasks in Indian households.

Adaptations in occupational activities are reflected in the study findings, such as scheduling works during cooler periods of the day. In Germany, workers adjust their routines and use informal practices like foot baths and keeping doors open to maintain airflow, illustrating the importance of flexible work arrangements and supportive workplace policies in enhancing heat resilience.^17^ Similarly, in military settings, acclimatization protocols are followed, with field-based activities performed during early hours and desk jobs during peak heat times. Additionally, the military protocols for heat stress in India, as detailed by the National Disaster Management Authority, include restriction of working hours, provision of spacious and well-ventilated living accommodations, ensuring at least 10 min of rest in each hour of walking, establishment of heat stroke centres and cool rooms, health education including training on first aid, organizing water drinking parades, implementing a buddy system for monitoring and support, ensuring adequate rest and sleep, promoting regular bathing and discouraging alcohol consumption.^18^

The effective dissemination of heat-related information in Rajasthan showcases the integration of modern communication methods with traditional community networks. Updates from the state Meteorological Department and official notices about impending heatwaves enable proactive adjustments, such as altering MNREGA working hours to protect labourers. Community groups like ‘Sojat’ play a crucial role in spreading awareness, leveraging social media and other media channels to ensure rapid information flow across villages. This grassroots approach ensures that even those without direct access to digital media are informed through interpersonal communication channels.

However, socio-economic disparities significantly impact the ability of individuals to cope with extreme heat. As noted by participants, access to cooling resources such as fans or air coolers is often limited by financial constraints. This highlights the need for targeted interventions to support vulnerable populations, ensuring equitable access to cooling solutions and reinforcing community resilience against heat stress.

While technological adaptations like air conditioning are often emphasized, our findings, along with those from other regions, reveal that sociocultural practices can be equally, if not more effective in certain contexts. Overreliance on air conditioning can impair physiological acclimatisation and increase vulnerability to heat.^19^ Therefore, integrating sociocultural practices with technological solutions provides a more balanced and sustainable approach to heat adaptation.

Participants reported traditional dietary practices largely centred on millets, pulses and dairy-based foods. Millets are particularly valued for their high resistance to heat and drought and superior nutritional profile compared with conventional cereals. Seasonal cultivation of crops is commonly practiced to optimize water use and maintain food security. Maize, legumes and pulses are widely grown due to their low water requirements and resistance to spoilage, and they form staples of the local diet, with maize roti being a frequently consumed item. Legumes and pulses are favoured not only for their nutritional benefits but also for their long shelf life, making them well-suited for storage under hot and arid conditions.

The government of India is actively working to upgrade primary and secondary health facilities, aiming to enhance healthcare delivery across the country. However, capacity building for human resource infrastructure remains limited, posing challenges to the effective implementation of health initiatives. The introduction of Health and Wellness Centres^20^ and the revisions to the Indian Public Health Standards^21,22^ are commendable steps towards improving health systems, offering a more structured and standardized approach to healthcare services. The National Disaster Management Authority, in collaboration with state authorities, has been proactive in developing comprehensive strategies to combat heat-related challenges. Heat action plans^23,24^ were made previously by the state government and institutions as a guide for implementation strategies.

In the context of environmental adaptation and resilience, as said by a participant, the Bishnoi movement stands as a historical example of community-driven conservation efforts in India. Originating in the 15th century, this movement is celebrated for its commitment to preserving biodiversity and combating environmental degradation. The Bishnoi community’s dedication is epitomized by the sacrifice of Amrita Devi (the person who led the protest) and 363 villagers who gave their lives to protect trees, demonstrating a profound respect for nature that remains relevant in today’s climate adaptation strategies.^25^

The study’s strengths include comprehensive data collection through various qualitative methods, a diverse participant pool providing holistic insights and context-specific findings that highlight traditional knowledge. The focus on vulnerable populations and practical, policy-relevant recommendations further enhances its significance. Additionally, capturing data from March to June, a period characterized by extreme heat, provides a robust understanding of heat adaptation practices.

Policymakers in Rajasthan could consider implementing targeted interventions to reduce heat vulnerability among socio-economically disadvantaged populations, such as providing subsidies or incentives for the purchase of cooling devices like fans and air coolers for below-poverty-line households. Additionally, integrating heat-resilient design principles into rural housing schemes, including the *Pradhan Mantri Awas Yojana Gramin*, and promoting the adoption of clean cooking fuels such as LPG to reduce indoor heat and air pollution could further enhance adaptive capacity. Measures such as air conditioning public transportation during peak summer periods may also mitigate heat exposure at the community level. Providing farmers with seeds of water- and heat-resistant crops could be another intervention. Future research should focus on identifying and evaluating cost-effective sociocultural and indigenous adaptation practices across diverse settings, thereby providing an evidence base for the development of comprehensive heat adaptation guidelines.

## Conclusions

The study reveals a diverse range of sociocultural practices and health-seeking behaviours in Rajasthan to cope with extreme heat. Integrating traditional methods like consuming tamarind water and using ORS with modern public health strategies can enhance heat resilience. Comparative insights from other regions also underscore the universal relevance of such practices. The study emphasizes the need for targeted interventions, particularly for vulnerable groups, and advocates for a balanced approach combining sociocultural practices with technological solutions after adequate scientific validation. The Rajasthan government’s initiative to establish heatwave wards in community health centres provides a proactive model for addressing heat-related health risks.

## Supplementary Material

ihaf153_Supplemental_Files

## Data Availability

The data used in the present study are available upon reasonable request to the corresponding author.
