# Supplementary material for: Sociocultural and indigenous practices of rural India in adapting to heat stress: an exploratory descriptive qualitative study
Source: Int Health. 2025 Dec 26;18(4):511–21. doi: 10.1093/inthealth/ihaf153 (PMC13329950; doi:10.1093/inthealth/ihaf153)
Supplement: ihaf153_Supplemental_Files [file ihaf153_supplemental_files.zip › HRI_Codes&Themes (for submission)_05.02.2025.pdf]

Verbatims and sub-themes based on the interviews and FGDs

Themes	Sub-themes	**Quotes (Verbatim)**
Perception about heat-related illnesses	Physical symptoms and misconceptions	• "I feel dizzy upon going in the hot sun." *(Adult female)*<br>• "Upon returning home from hot sun, we feel nauseating, dizziness, weakness. We tend to lie down on the cot for the rest of the day, with wet clothes upon us. Elders at home used to do this." *(Adult male, who is a farmer)*<br>• "With increase in heat, a person may feel jitters ("ghabrahat/घबराहट"), dizzy, weak and tired." *(Adult female at household)*<br>• "In children, we have observed that they experience fever-like symptoms after outside play, and vomiting. And when you go to the doctor, they give you ORS powder to drink." *(An old female in the household)*<br>• "*Cholera disease (diarrhoea)* is very common among the old persons. If there is no arrangement for cooler or A/C for them, then it becomes difficult for them even for a day, and sometimes it even leads to death if they go out of the house." *(Anganwadi helper)*<br>• "We have not heard anything like this, neither in the village nor in the surrounding areas, people were sick because of *corona virus* but not because of the heat." *(Adult male working in a shop)*<br>• "There has been no death because of heat in the village in the recent years." *(Young adult male)*<br>• "I had heard from my parents that people died due to heat, but have never seen it in the neighbourhood or among relatives." *(Adult male in the Panchayat office)*
	Home remedies	• "We do local treatment at home, give tamarind water if they are tired and exhausted. If they do not recover within some time, we take them to the hospital."<br>• "In earlier days, when there was anybody who was affected with heat illness, we used to apply "*multani mitti*" (Bentonite's clay) over the body, along with a ground bitter-gourd drink, if they are conscious." *(An aged male villager)*<br>• "Orally, we give fluids like the buttermilk, 'lassi' (curd with salt or sugar), boiled vegetable water, lemon juice."<br>• "We make decoction by adding sugar candy, coriander, black pepper and cloves." *(Old female in the household)*<br>• "Applying wet cotton cloth over the body as a remedial measure."<br>• "Giving lemon water, giving ORS solution and using curd. If even after this the temperature is not decreasing, then we take to the hospital." *(ASHA in the village)*<br>• "As soon as lemonade is given, give cold water with *Amla.*"<br>• "Due to heat, there are remedies at home like we have curd and buttermilk, we have tamarind water, we drink it by mixing ice cubes, these are only home remedies. If there is more problem, we then go to the hospital." *(Village sarpanch)*

Verbatims and sub-themes based on the interviews and FGDs

Themes	Sub-themes	**Quotes (Verbatim)**
	Awareness about heatwaves and alerts	• "The temperature is getting higher, earlier we did not perceive heat this much, but now it is rapidly increasing. This is happening due to the climate change, and pollution." *(An aged female in village)*<br>• "The heat here is increasing continuously, the heat is getting perceived more than before. Even in the summer season, it is raining in the middle, and then it gets hot again. These phenomenon are not experienced every time."<br>• "It has been getting hotter for the last three-four years. The increasing heat is also affecting our food habits, drinking water, and diseases are on the rise as well."<br>• "Information is received four-five times in 12 months whenever a meeting is held in the village."<br>• "It comes in the newspaper but we do not read it."<br>• "We feel that heat wave is going on, apart from this, at some places, we see in newspapers, on phone or on TV and read that heat wave is going on."<br>• "We don't know that, we don't have a smartphone."<br>• "Nowadays, people receive information about the temperature and impending hot air through multimedia phones. This information is also available in newspapers."<br>• "This hot air is noticeable to people, and information about it is widely available. We get updates from the Rajasthan Meteorological Department and receive official notices predicting increased heat in the next few days. In response, we adjust our MNREGA working hours accordingly. We also inform village residents to stay hydrated during heat waves. We have a group named ***'Sojat'*** to disseminate information to our gram panchayat. With social media, everyone is quickly informed. For instance, if a young person sees a weather update at home, they inform everyone. We also rely on TV channels and other media for updates, and people often share this information through statuses and messages."

Verbatims and sub-themes based on the interviews and FGDs

Themes	Sub-themes	**Quotes (Verbatim)**
Environmental Adaptations and Practices	Housing Adaptations	• “Earlier, when we lived in the kaccha houses, the roofs were made of mud and hay, which kept the house cooler than the outside environment. Nowadays, we build pucca houses with concrete roofs and no windows, then there is no ventilation. Only if there is a fan or a cooler, it seems fine.” *(An old person in the village)*<br>• "Earlier the roofs were separate and the houses were kaccha, due to which it remained cool in the summer months. Now the roofs are made of tin sheets, which get hot quickly, and are also being made of concrete, making it a closed structure.”<br>• "If you have a thatched roof, open windows, coat the roof with limestone or alum, keep the house clean and mop it regularly, it remains cool.”<br>• “We install ventilators/exhausts for air flow and keep windows open in the house” *(Adult male)*<br>• “We use air coolers at our home, there are 3 coolers, one in each room.”<br>• “We use coolers and fans. We also have windows in every room and solar panels installed over the roof. The house remains cooler than the outside.” *(Village sarpanch)*<br>• "In summer, we soak sacks or similar materials in water, hang them over the entrance and the windows, such that it prevents hot air from entering the house."<br>• **“**We install stoves in the setback of the house, in the open.”<br>• "We cook food very early in the morning, and in the evening, to avoid hot air from the kitchen inside the house during hotter hours of the day.”<br>• "Panchayat gets tents set up and the villagers also make huts and install water tanks and as MNREGA runs, they also put up tents there.”
	Protection of Cattle	• "We tie the cattle in the shade under trees within the house."<br>• "We make *‘Chhapra’* (made of grass and thatch) for them because it remains cooler.” (Adult female farmer)<br>• "There are no facilities for those animals that live outside on the streets. At home we take care of our animals."<br>• "Here, the people of the Bishnoi community are deeply devoted to wildlife. Additionally, they take measures such as spraying water on animals and using cooler fans. They also wet sacks and place them nearby to prevent the animals from getting hot air, and they sprinkle water on the thatched roofs as well.” (Adult male in the village administration office)<br>• "The village administration does not have much role in this; the villagers themselves organize and create water sources in public places for animals to drink. There are frequent power cuts here, lasting two to three hours every day."
	Environmental Protection	• "The trees and plants are being cut, so it is natural that the temperature will rise."<br>• "I have seen in the last 5 years the temperature gradually increasing. The villagers have also planted saplings here, so that the heat can be reduced a little."<br>• “Only a few people are interested to plant trees, while others do not participate in it. Last year, the Gram Panchayat had given medicinal plants to every house. It also feels good that you can breathe and experience cool air by sitting under the plant.”

Verbatims and sub-themes based on the interviews and FGDs

Themes	Sub-themes	**Quotes (Verbatim)**
Changes in lifestyle and daily activities	Vocational Adjustments	• "We wake up early in the morning and go to work, come back by noon, then sleep till 2-3 o'clock and then come back to work in the late afternoon." *(Adult female employed as MNREGA worker)*<br>• "We *(MNREGA workers)* begin our work early in the morning and continue until 11 o'clock." *(Adult male employed as MNREGA worker)*<br>• "In the summer season, if the regular work time is 8 hours, it is reduced to 4-6 hours. Similarly, the working hours for MNREGA workers are also nearly halved. For instance, if the normal schedule is from 9 AM to 5 PM, it is adjusted to run from 6 AM to 11AM or 12PM." *(Supervisor at MNREGA work)*<br>• "Everyone gets up at five o'clock and goes to work." *(Aged male farmer)*<br>• "After completing our work early in the morning, we sleep in the afternoon, and our sleep gets balanced." *(Anganwadi worker)*<br>• "We used to work for 12 hours, now in summer we are not able to work more than six hours." *(A manual labourer)*<br>• "If it is not hot, we work the whole day. If it is hot, we rest for 2 to 4 hours in the afternoon." *(ASHA in the village)*<br>• "Farming cannot be done properly during hotter months. Workers get easily tired due to the high temperature, so we need to hire more workers to complete the work on time."<br>• "If people don't work for 15 days in a month, they can't sustain their livelihood, so they need to work continuously. Only if they work today, the money they earn will support their household tomorrow, meaning they must work even in the summer. These individuals labor under the sun. To cope with this, they take measures to minimise exposure to the sun, such as reducing their time in direct sunlight to 5 to 6 hours. Apart from that, they only drink cold water to stay cool."<br>• "People here have to go to work due to compulsion."<br>• "It affects daily life as a labourer. Those who go to manual work are hesitant to go to work due to heat. labourers get more tired due to heat. They experience fever-like symptoms in the evenings, so they go to work less, thereby affecting their income. In colder weather, they can work continuously for three to four hours, but with the current heat, they struggle to work for even 15 minutes at a stretch."

Verbatims and sub-themes based on the interviews and FGDs

Themes	Sub-themes	**Quotes (Verbatim)**
	Food and Water Practices	• "Take more of raita made from curd, make *sangri* (desert bean), eat green vegetables, especially green leafy vegetables like spinach."<br>• "Green vegetables are available here, and dry vegetables are also used like *"Ker Sangri"* (a dish made of desert bean and capers). Apart from this, *rabdi* (a dessert made of thickened milk) made at home, is also consumed."<br>• "In the summer season, onions are primarily used here because they help cure many diseases and benefit the body. People consume more onions, buttermilk, rabri (condensed milk based dish), curd, and millet roti during this time."<br>• "They give millet porridge and ladoos, and drink 'bangh' during Holi."<br>• "We do not eat hot food like hot spices, fried legumes etc."<br>• "It is preferred to consume less of fried foods during summer."<br>• "We eat very little oil during summers."<br>• "In the village, we eat these water containing fruits like watermelon and melon during summer."<br>• "We eat mango, banana, papaya, watermelon." *(Adult female in the household)*<br>• "Millet roti. Vegetables, boiled pulses and legumes and like milk is curd and buttermilk, curd is taken more in summer."<br>• "Sugar is soaked in water and consumed. "Kevala flowers" (screw pine) are kept soaked in a cold pot, it is considered to cool down the body."<br>• "In the summer season, we also use tamarind, soaking it overnight and drinking the water it soaks in, as well as applying it on our feet. Additionally, we consume sugar and use the leaves and flowers of *'Kevala'* (screw pine). We soak *'kevala'* in water, use its juice, and even bathe with it."
	Transportation	• "We carry a water bottle, we carry a towel, we carry a carpet."<br>• "A plastic bottle is covered with cloth and soaked in water, which keeps it cool. In the summer season, most people use '*matki*' pots (earthen pots) for storing water, while in winter, steel urns are used more frequently."<br>• "We cover the water bottles with wet cloth (*jute*)."<br>• "Wear cotton clothes and according to their needs and affordability, some can carry an umbrella, while others travel in a car."

Verbatims and sub-themes based on the interviews and FGDs

Themes	Sub-themes	**Quotes (Verbatim)**
System-level and Environmental Challenges	Water and Power Supply Issues	• "There are elections coming up, hence water will be made available, otherwise we do not get regular supply. We hire water tankers, at least two every month. Water is made available once in 10-15 days, sometimes we get it only for one or two hours in a day through the tap. There are also issues with electricity, due to which we are not able to operate the pumping motors and fill waters in the storage tanks. Long hours of electricity cut makes it even more harder to store water in adequacy.”<br>• "Trees and plants are planted by the village administration, but they are not maintained. We even sent photos to the government. We should do the cleaning ourselves, make arrangements for water and electricity.”<br>• "There are usually water problems in summers because of increased need. There is also power cut for four-five hours in a day, making it difficult to store water.”<br>• "There is a problem of electricity cut, almost 5 to 7 times in the whole night."<br>• "Electricity should be made available properly. There are many power cuts here."<br>• "In June, there is a bit of a water problem because the water supply from the water body (lake) is cut off. As a result, the government provides water through tankers.”
	Issues with Healthcare Services	• “When we go to the hospital, they don’t listen very well about the history, they give medicines for fever and send us away."<br>• “No doctor is available to attend to us after 1 PM, so we have to take the patient farther to a private hospital.”<br>• “Doctors stay in the hospital only until 12:00 PM in summers, and till 2-3 PM in winter. It is open as long as they are available, after which only one nursing staff is available, who doesn’t know much about managing these illnesses” (Anganwadi worker)<br>• “There are very limited facilities available here, we usually have to take the patient to the city if there is an emergency” (ASHA in the village)<br>• “In the hospitals, they refuse to admit the patient if we go in very sick, when home remedies haven’t worked.” (Adult female)<br>• “The prescribed medicines are usually not available at the PHCs, we need to buy from outside.” (Adult male)<br>• “No field workers come for conducting surveys, or providing medicines”
