# Supplementary figures and images for: Sociocultural and indigenous practices of rural India in adapting to heat stress: an exploratory descriptive qualitative study
Source: Int Health. 2025 Dec 26;18(4):511–21. doi: 10.1093/inthealth/ihaf153 (PMC13329950; doi:10.1093/inthealth/ihaf153)

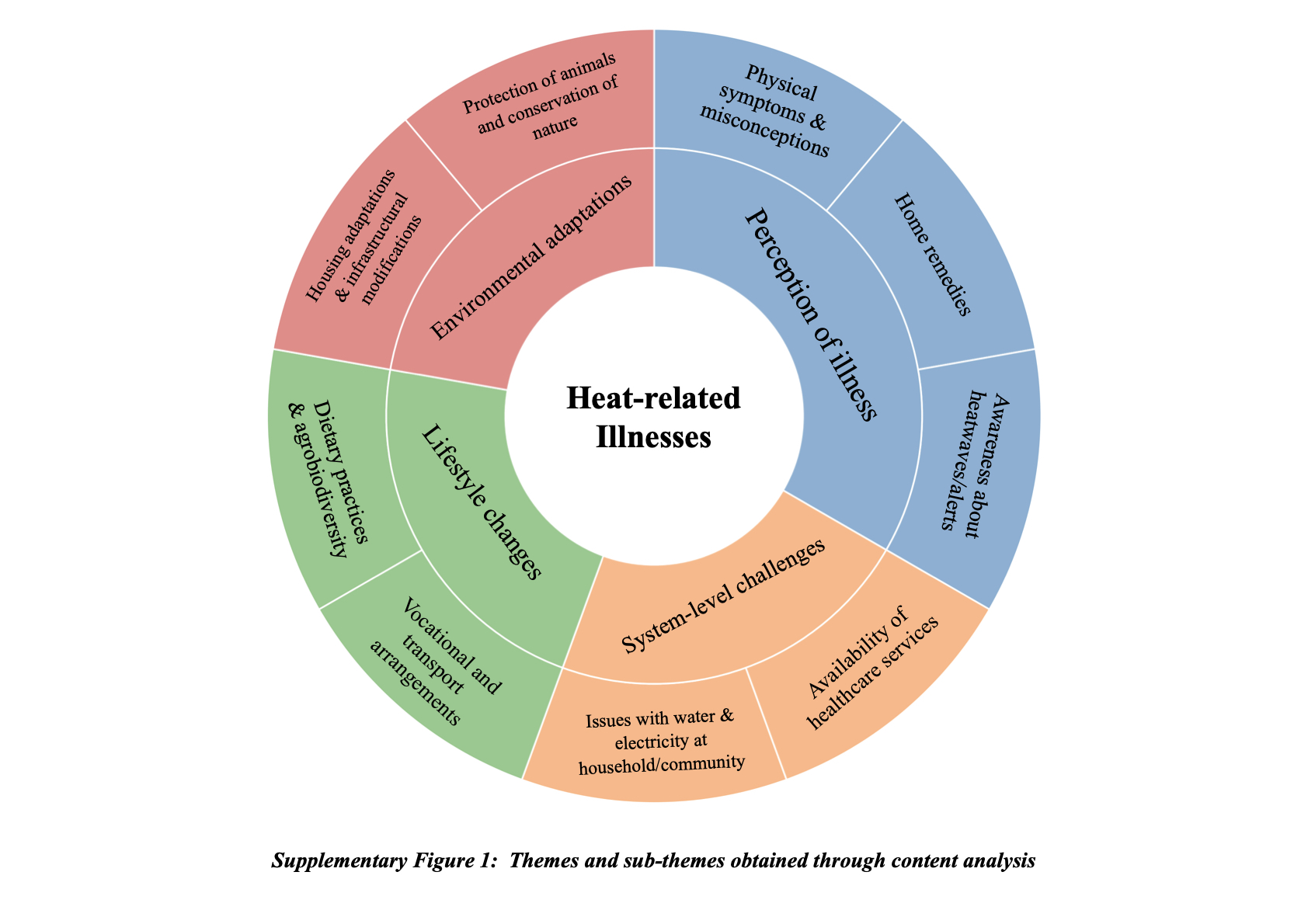

Supplement: ihaf153_Supplemental_Files [file ihaf153_supplemental_files.zip › Sunburst_HRI.1.jpeg]
